# Pulmonary Involvement in Patients with Positive Myositis Antibodies in Rheumatology: A Retrospective Monocentric Analysis

**DOI:** 10.3390/jcm14155443

**Published:** 2025-08-01

**Authors:** Falk Schumacher, Malte Kanbach, Maximilian Zimmermann, Daniel Majorski, Wigbert Schulze, Maximilian Wollsching-Strobel, Doreen Kroppen, Sarah Bettina Stanzel, Wolfram Windisch, Johannes Strunk, Melanie Berger

**Affiliations:** 1 Department of Rheumatology, Krankenhaus Porz am Rhein, 51149 Cologne, Germany; 2 Faculty of Health, School of Medicine, Witten/Herdecke University, 58455 Witten, Germany; 3 Department of Pneumology, Kliniken der Stadt Köln, 51109 Cologne, Germany; 4 Wisplinghoff Medical Laboratories, 50858 Cologne, Germany

**Keywords:** myositis, idiopathic inflammatory myopathy, lung involvement, interstitial lung disease, myositis-specific antibodies, myositis-associated antibodies, pulmonary function test, high-resolution computed tomography

## Abstract

**Background:** Pulmonary involvement is the most common prognosis-related organ involvement in idiopathic inflammatory myopathy (IIM). Owing to the large number of antibodies, the evidence for lung involvement and rare antibodies is limited. In everyday clinical practice, the interpretation of positive myositis antibodies represents a challenge. **Methods:** This study is a retrospective monocentric analysis. The data collection regarding positive myositis antibodies and possible pulmonary involvement was carried out from July 2019 to May 2022. Data analysis revealed positive results for one of the following antibodies: EJ, PL7, OJ, PL12, Mi-2α, TIF1γ, MDA5, SAE, NXP2, SRP, Ku, PM-Scl100 and PM-Scl75. In our analysis, patients with IIM, patients with inflammatory rheumatic disease other than IIM and patients without inflammatory rheumatic disease are described. The results of high-resolution computed tomography (HRCT), pulmonary function tests, echocardiographic examinations and their associated clinical findings are examined. **Results:** In the entire cohort, 209 patients with positive myositis antibodies were detected. In total, 22 (10.5%) patients had interstitial lung disease (ILD) patterns on HRCT. In the subgroup of patients with IIM, a significantly higher proportion of patients with lung involvement (*n* = 13, 35.1%) was found than in the group with other inflammatory rheumatic diseases (IRDs) (*n* = 6, 6.7%) or in the group without IRDs (*n* = 3, 3.7%). When the antibody groups were considered, the PL12-positive patients had the largest proportion of ILD (42%), followed by the MDA5-positive patients (40%). **Conclusions:** In patients with IIM, myositis antibodies are highly relevant for assessing the risk of lung involvement. In groups with other IRD or without IRD, antibody detection does not represent this high relevance for lung involvement. A differentiated assessment of the various MSAs or MAAs detected, as well as clinical parameters, allows for further important risk assessment for prognosis-relevant lung involvement.

## 1. Introduction

In rheumatology, the prognosis and treatment of patients largely depend on organ involvement. In idiopathic inflammatory myopathy (IIM), pulmonary involvement plays a major role because of its frequency and prognostic relevance [1]. Only very limited data are available regarding pulmonary involvement with rare positive myositis antibodies in IIM patients. Due to a lack of evidence for rarer antibodies, only Jo-1 antibodies are included in the ACR-EULAR classification criteria of the IIM [2]. This highlights the unmet need for evidence in this area. Furthermore, there are insufficient data on the clinical relevance of positive myositis antibodies in groups of other IRDs or in patients without IRDs. However, the significance of positive myositis antibodies in the various disease groups represents a significant challenge in the clinical practice of rheumatology. A detailed examination of the clinical phenotypes of the three diagnostic groups described above in one study, such as is carried out in this work, does not yet exist.

IIMs are a heterogeneous group of inflammatory rheumatic diseases (IRDs). On the basis of their different clinical manifestations and extended antibody diagnostics, a differentiated classification of the group of IIMs into antisynthetase syndrome (ASS), dermatomyositis (DM), polymyositis (PM), overlap myositis (OM), immune-mediated necrotizing myopathy (IMNM) and inclusion body myositis (IBM) is possible [2,3]. Depending on assignment to an IIM subgroup and antibody status, a heterogeneous picture with different clinical manifestations also emerges in the group of patients with IIMs and lung involvement. In other cohorts of IIM patients, interstitial lung disease (ILD) occurs in approximately one-third of patients. It is important to highlight the increased incidence of ILD, especially in association with ASyS [4].

An analysis of lung involvement in relation to various antibodies can reveal regional differences. For example, in Asia significantly higher rates of progressive lung involvement in MDA5-associated IIMs have been described than in Europe [2,5]. Furthermore, the pretest probability of antibody determination can, of course, also be influenced by the experience of the various centres and various relevant specialist departments. Also, different laboratory tests are available in each region, which are not always easily comparable.

Pulmonary involvement can appear in all subsets of IIMs but is more frequent in ASyS, DM and OM, in which the frequency depends largely on the specific antibody. Because of its prognostic relevance, lung involvement in IIM is often focused on ILD. However, it is important to observe other forms of lung changes, such as pulmonary arterial hypertension (PAH), pleural disease, infections, drug-induced toxicity, malignancy (cancer-associated myositis (CAM)) and respiratory muscle weakness [6].

High-resolution computed tomography (HRCT) is a very important diagnostic tool for ILD. However, image morphological changes in the context of IIM can be very different. The patterns of organizing pneumonia (OP) and nonspecific interstitial pneumonia (NSIP) are more common than the pattern of usual interstitial pneumonia (UIP) [7]. Consolidating lung changes on HRCT, such as OP, are often described in the setting of acute lung changes in IIMs. In cases of early therapy, a favourable outcome regarding these lung changes is observed [8,9].

Pulmonary function testing (PFT), aimed at recording a baseline value and quantifying disease severity with the forced expiratory vital capacity (FVC), total lung capacity (TLC) and diffusion capacity of carbon monoxide (DLCO), is part of the standard diagnostic method. Before the availability of HRCT, the DLCO in combination with a conventional thoracic X-ray examination was also suggested to be suitable for screening for connective tissue disease (CTD)-ILD [10]. However, PFT is not suitable as the sole screening tool for detecting ILD in everyday clinical practice. In addition, other studies have shown that PFTs are useful for monitoring the course of an ILD. FVC and DLCO have prognostic significance with regard to mortality in idiopathic pulmonary fibrosis patients [11]. A retrospective study of ASyS-ILD patients treated with prednisolone revealed that the PFT measured by the DLCO and by the FVC is maintained and even improves. In the presence of anti-Jo-1 antibodies the improvement in the DLCO was significantly higher compared to that in the presence of anti-PL-7, anti-EJ and anti-PL-12 antibodies [12].

Pulmonary involvement in IIMs can occur before, at the same time as or after the onset of other symptoms of IIMs. Therefore, a closer look at the connection between certain myositis antibodies and pulmonary manifestations in patients with IIMs is important to draw possible conclusions in the case of positive myositis antibodies in other IRDs or in patients without a diagnosis of inflammatory rheumatic disease.

### 1.1. Anti-tRNA-Synthetase Autoantibody-Associated Lung Involvement in IIM

The Jo-1 antibodies form by far the largest group of antisynthetase antibodies in terms of their frequency. In this group, a frequency of ILD of 66% has been described in the literature. With regard to PL7 and PL12 antibodies, an even greater proportion of patients with IIM and ILD (84%) has been described [6]. With respect to the other available antisynthetase antibodies, very few data exist regarding lung involvement in IIM.

### 1.2. Dermatomyositis-Specific Autoantibodies and Lung Involvement in IIM

Notably, regarding the antibodies associated with dermatomyositis, in the Mi-2, NXP-2, SAE and TIF1γ groups, a relatively low frequency of ILD in IIM patients has been described in the literature. With respect to MDA5 antibodies, there is a significantly greater frequency of ILD. The frequency of ILD occurrence in the context of MDA5-positive dermatomyositis varies locally. In Asia, the frequency is slightly higher at 90% than it is in Europe at 60%. The rapid progression of ILD in MDA5-positive IIM patients is a very important prognostic feature in this group and is now well described in the literature [2,5].

### 1.3. Immune-Mediated Necrotizing Myopathy (IMNM)-Associated Antibodies and Lung Involvement in IIM

The IMNM group often includes antibodies against 3-hydroxy-3-methylglutaryl-coenzyme A reductase (HMGCR) or signal recognition particle (SRP). Statins predispose patients to the development of this myopathy through the upregulation of HMGCR [13]. Extramuscular manifestations are rather rare in anti-HMGCR-IMNM.

With respect to detected SRP antibodies, increased cases of ILD in the context of IIM have been described in the literature, although at 15%, these cases are rather less common than those in other groups. In this group, the focus is on severe myositis, although unfortunately, this group has thus far shown a relatively poor response to immunomodulatory therapy [14].

### 1.4. Myositis-Associated Autoantibodies (MAAs) and Lung Involvement in IIM

In other cohorts, a frequency of ILD in patients with IIM and PM-SCL antibodies of 38% has been described, with the occurrence of pulmonary symptoms before the appearance of skin changes or myopathy being lower in the PM-SCL antibody group than in the group with antisysthetase antibodies [7,15,16]. The group of patients in the Ku antibody group also had a high incidence of ILD (38%). There is often an overlap syndrome consisting of myositis and systemic sclerosis [17,18,19].

## 2. Materials and Methods

### 2.1. Study Design

This study is a retrospective monocentric analysis. The data collection regarding positive myositis antibodies and possible pulmonary involvement was carried out as part of our register, which was created in 2022 and lists all patients for whom a myositis antibody was requested by a rheumatologist [20]. The collection of data was carried out without personal data in pseudonymized form and follows the Declaration of Helsinki. The study was approved by the ethics committee at the University of Witten/Herdecke, Germany (Ref: 153/2022). In this review the ethics committee did not see any need for consent to participate. A retrospective monocentric analysis of all orders to determine MSAs or MAAs in the period from July 2019 to May 2022 in the inpatient and outpatient sectors in the Department of Rheumatology, Krankenhaus Porz am Rhein, Cologne, Germany, was carried out for this study. All documented information regarding pulmonary involvement in this group was evaluated.

### 2.2. Laboratory Tests

All results for the MSAs and MAAs included in this analysis were determined by an external laboratory (Wisplinghoff Medical Laboratories, Cologne, Germany). Sera were tested with the Euroline Autoimmune Inflammatory Myopathies 16 Ag IgG-Immunoblot (Euroimmun, Lübeck, Germany), coated with the following 16 MSA and MAA antigens: Mi-2α, Mi-2ß, TIF1γ, MDA5, NXP2, SAE1, Ku, PM-Scl100, PM-Scl75, Jo-1, SRP, PL7, PL12, EJ, OJ, Ro52 and control. For this study the following antigens were included for the evaluation process: EJ, PL7, OJ, PL12, Mi-2α, TIF1γ, MDA5, SAE, NXP2, SRP, Ku, PM-Scl100 and PM-Scl75. Jo-1 was not included because the focus of this study was on the analysis of patient cases with less common myositis antibodies, as the evidence in this area is particularly lacking. Unfortunately, anti-HMGCR antibodies are very rarely detected in the clinical practice of our centre because they are not included in standard myositis panels. Therefore, we did not have sufficient data at our centre to include them in the study.

### 2.3. Collected Patient Data

For all patients with positive detection of one of the 13 antibodies described above, the patient files were analysed for lung involvement. Analysis of demographic data and the main rheumatological diagnoses and a detailed and systematic analysis of the symptoms that were documented were also performed.

Symptoms that could be evaluated as part of a skin manifestation in the context of an IIM were documented for each patient. IIM-related skin changes include rash, Gottron signs and papules, heliotrope rash, pruritus, alopecia, peripheral oedema, mechanic’s hands, puffy hands, digital ulcers, periungual telangiectasias and sclerodactyly. In addition, the occurrence of Raynaud’s syndrome, myalgia/muscle weakness, arthralgia, arthritis, dyspnoea at rest/on exertion, fever > 38 °C, fatigue and weight loss was analysed via the available data.

### 2.4. Diagnostic Groups

For further evaluation of the data, the patients were divided into three diagnostic groups: IIM, other IRD and no IRD. The first group, IIM, includes antisynthetase syndrome (ASyS), dermatomyositis (DM), polymyositis (PM), overlap myositis (OM) and immune-mediated necrotizing myopathy (IMNM). Overlap myositis (OM) was defined as myositis in the context of another IRD, such as systemic lupus erythematosus (SLE), systemic sclerosis, undifferentiated connective tissue disease (UCTD), mixed connective tissue disease (MCTD) or Sjögren’s syndrome.

The second group, other IRD, was defined on the basis of the following diagnoses without overlap to myositis: systemic sclerosis (SSc), systemic lupus erythematosus (SLE), undifferentiated connective tissue disease (UCTD), rheumatoid arthritis (RA), spondyloarthritis (SPA) and other IRDs such as giant cell arteritis, cryoglobulinaemic vasculitis, Behçet’s disease, granulomatosis with polyangiitis, microscopic polyangiitis, eosinophilic granulomatosis with polyangiitis, primary Sjogren syndrome, mixed connective tissue disease and interstitial pneumonia with autoimmune features. All the other main diagnoses were assigned to the group with no IRD.

### 2.5. Analysed Parameters Related to Lung Involvement

In addition to the evaluation of the HRCT images, the data from the PFT, information on the occurrence of pleurisy and data on the probability of PAH were also analysed to provide a differentiated picture of the various possible areas of lung involvement in our cohort.

For each patient, whether one of the lung function parameters examined was abnormal was documented. The abnormal values of PFT were defined as TLC < 80% predicted, FVC < 80% predicted and DLCO < 80% predicted. The first available PFT after the initial presentation in the rheumatology department was documented. The presence of pleurisy was evaluated based on the descriptions in CT chest images or sonography findings. A systolic pulmonary arterial pressure of >25 mmHg on transthoracic echocardiography (TTE) was defined as a possible indication of PAH.

The analysis of the HRCT findings for the thorax was carried out according to the findings, which were collected via an interdisciplinary consensus of rheumatologists, pulmonologists and radiologists. All available HRCTs of the respective patients were included in the analysis, and in the first step, it was determined whether a relevant ILD pattern could be detected by HRCT at all. In the second step, all abnormal HRCT images were re-evaluated regarding the existing pattern. The categorization of NSIP, UIP and OP by the interdisciplinary team also took place. In the case of overlapping image morphological findings, the predominant pattern was determined for analysis. This procedure was repeated by a second independent ILD board blinded to the findings of the first group. Inter-observer disagreements were resolved by consensus.

### 2.6. Statistical Analysis

Owing to the heterogeneous group described here, the descriptive presentation of the data was placed at the forefront of the analysis. The data were described by measures of central tendency (mean) and dispersion (standard deviation (SD)). Statistical analyses and figures were generated via Microsoft Excel version 2307 (Microsoft, Redmond, WA, USA). Records with missing or unknown data for any variables of interest were excluded from the analyses.

## 3. Results

### 3.1. Patient Demographics and Characteristics

During the time interval from July 2017 to May 2022, 209 different patients were found to be positive for MSAs or MAAs (EJ, PL7, OJ, PL12, Mi-2, TIF1γ, MDA5, SAE, NXP2, SRP, Ku, PM-Scl100 and PM-Scl75).

Sixty-nine percent of the 209 examined patients were female. The mean (SD) age was 60.5 (±5.6) years. In 92 (44.2%) of these patients, an HRCT chest scan was performed based on clinical indications. Interstitial lung changes were observed in 22 patients (10.5%).

The patients were divided into three diagnostic groups. In these groups, different proportions of patients with ILD evidence on HRCT can be detected. Evidence of ILD was clearly more common in the IIM subgroup (35.1%) than in the other groups (Table 1). A detailed examination of the various IIMs revealed that the frequency of ILD occurrence in the context of polymyositis (14%) is lower than that in the context of ASyS, DM or OM. In the group of patients with myositis antibodies and a diagnosis of another IRD other than IIM, the largest proportion of patients with an ILD indication on HRCT was found in the group of patients with systemic sclerosis (40%). A detailed evaluation of the diagnostic groups and the various manifestations of lung involvement are shown in Supplementary Figure S1.

### 3.2. Lung Involvement in the Different Antibody Groups

In our analysis, patients with idiopathic inflammatory myopathy (IIM), patients with an inflammatory rheumatic disease other than IIM (other IRD) and patients without inflammatory rheumatic disease (no IRD) were described. The analyses based on the entire cohort therefore do not differentiate between these groups.

**Figure 1:**
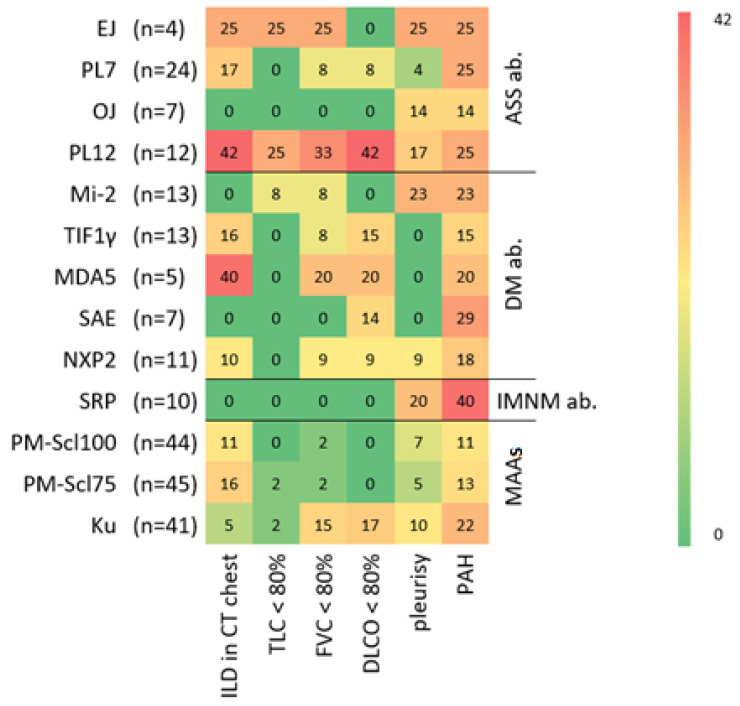
Abnormal examinations and manifestations related to lung involvement in the antibody subgroups. The *x*-axis shows the various tests documented for each patient. The *y*-axis shows the respective antibody groups. The numbers of patients within each subgroup are indicated on the right of each antibody. The proportion of patients in the various antibody subgroups who showed abnormal findings in the respective tests was visualized using a heat map. The numbers in boxes indicate the percentage of patients positive for a particular diagnostic test or manifestation in an antibody subgroup. The proportions are color-coded from green (0%) to red (42%). On the right edge of the heat map, the antibodies are assigned to the different groups of myositis antibodies. ASS ab.: antisynthetase syndrome-related antibodies; DM ab.: dermatomyositis-related antibodies; IMNM ab.: immune-mediated necrotizing myopathy-related antibodies; MAAs: myositis-associated antibodies. (ILD: interstitial lung disease; CT: computed tomography; TLC: total lung capacity; FVC: forced vital capacity; DLCO: diffusing capacity of the lungs for carbon monoxide; PAH: pulmonary arterial hypertension; ab.: antibodies).

The proportion of patients who were positive for PL12 antibodies and ILD was the highest in the whole cohort, at 42% (Figure 1). HRCT also revealed a relevant proportion of ILD in the EJ and PL7 antibody groups. Patients with antisynthetase antibodies and lung involvement on HRCT also mostly show abnormal values in the PFT. In all ASyS groups, there were patients with evidence of increased PAP values in echocardiography. The groups of PL7, PL12 and EJ antibodies were detected more frequently (each 25%) than the OJ group (14%).

In the group of patients with antibodies associated with dermatomyositis, the highest proportion of patients with ILD was found in the MDA5-positive group (40%). The examination of patients who were positive for SAE antibodies revealed an increased proportion of patients with abnormal values in the TTE regarding PAH (29%).

The group of patients examined with SRP antibodies, which are classified as associated with IMNM, showed no evidence of ILD on HRCT or PFT. Compared with all the other antibody groups examined, this group had the highest proportion of patients with evidence of PAH (40%) in the whole cohort.

The examined patients with evidence of MAAs represented the largest group of the cohort (*n* = 130, 62%). Compared with ASyS antibodies (0–42%) or DM antibodies (0–40%), there was a lower proportion of ILD patients on HRCT in this group (5–16%). Compared with the other MAA groups, the Ku antibody group presented the lowest proportion of ILD in the HRCT at 5%, with the highest proportion of abnormal values related to PAH occurring in this subgroup (22%).

### 3.3. Lung Involvement in Different Symptom Groups

**Figure 2:**
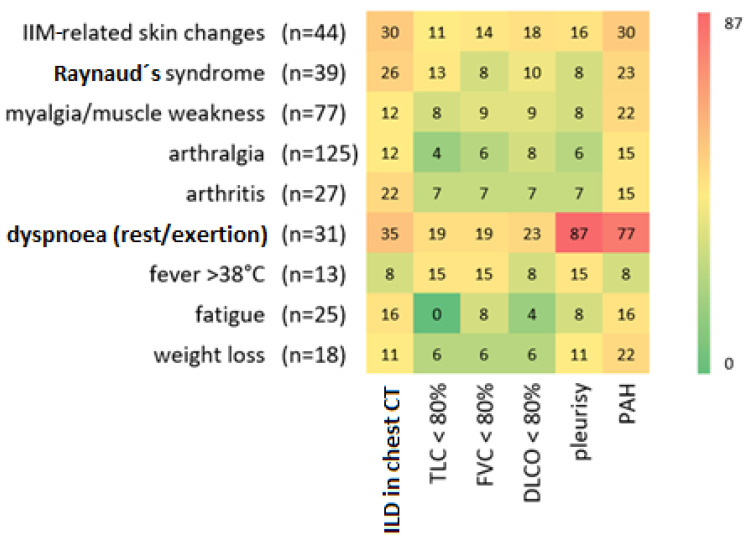
Abnormal examinations and manifestations related to lung involvement in the symptom subgroups. The *x*-axis shows the various tests documented for each patient. The *y*-axis shows the respective symptom subgroup. The numbers of patients within each subgroup are indicated to the right of the symptom. The proportion of patients in the various symptom subgroups who showed abnormal findings in the respective tests was visualized using a heat map. The numbers in boxes indicate the percentage of patients positive for a particular diagnostic test or manifestation in a symptom subgroup. The proportions are color-coded from green (0%) to red (87%). (IIM: idiopathic inflammatory myopathy; ILD: interstitial lung disease; CT: computed tomography; TLC: total lung capacity; FVC: forced vital capacity; DLCO: diffusing capacity of the lungs for carbon monoxide; PAH: pulmonary arterial hypertension).

If we divide our cohort into different symptom groups, differences can also be seen in the frequency of occurrence of lung involvement (Figure 2). The symptoms of IIM-related skin changes and dyspnoea at rest/on exertion represented an increased proportion of all examined parameters of lung involvement. A closer look at the evidence of ILD reveals that the groups with described Raynaud’s syndrome (26%) or evidence of arthritis (22%) are also associated with an increased proportion of ILD in HRCT. With respect to the PFT parameters, no clear trends can be determined regarding the individual symptom groups. However, the DLCO had the highest proportion of abnormal values in the dyspnoea group (23%). A positive finding for pleurisy was the most common positive finding for lung involvement in the group of patients with dyspnoea (87%). In comparison, the proportion of patients with dyspnoea in the group of patients with ILD on HRCT was rather low at 35%.

An increased occurrence of PAH is also evident in symptom groups other than dyspnoea (77%): IIM-related skin changes (30%), Raynaud’s syndrome (32%), myalgia/muscle weakness and weight loss (22%).

### 3.4. Myositis Antibodies and HRCT Patterns

**Figure 3:**
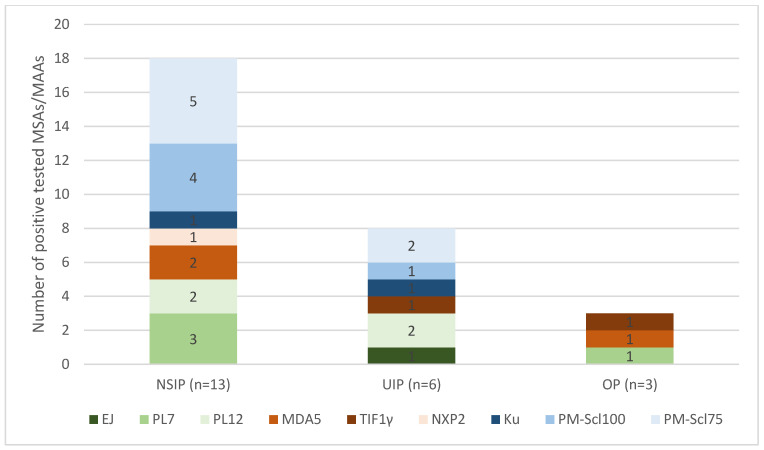
Distribution of myositis-specific antibodies (MSAs) and myositis-associated antibodies (MAAs) and their absolute levels of detection in the groups of CT patterns. The three different CT patterns, NSIP, UIP and OP, are shown along the *x*-axis in three columns. Next to the CT pattern name, the absolute number of patients in whom the respective CT pattern was detected is shown. The *y*-axis shows the number of patients in each CT pattern group. The absolute number of patients with the respective detected antibodies in each individual CT pattern group is color-coded as indicated in the legend. The size of the respective bar and the number indicated in the coloured bars indicate the absolute number of patients with the respective antibodies. (NSIP: nonspecific interstitial pneumonia; UIP: usual interstitial pneumonia; OP: organizing pneumonia).

A closer look at the 22 patients in our cohort who had proven ILD on HRCT also revealed differences in the HRCT pattern. The most common occurrence in this group was the NSIP pattern (*n* = 13, 59.1%). The occurrence of a UIP pattern is also evident in a relevant proportion (*n* = 6, 27.3%), and OP was detected in only a smaller proportion, *n* = 3 (13.6%). There were different proportions of detected MSAs and MAAs in the HRCT pattern groups (Figure 3). SAE, OJ and SRP antibodies were not detected in the group with ILD detected via HRCT.

In the group of 13 patients with an NSIP pattern, 18 different myositis antibodies could be detected. The MAAs are the largest group here (*n* = 10, 55.6%). The ASyS antibodies represented the second-largest group (*n* = 5, 27.8%), and the DM antibodies represented the smallest group (*n* = 3, 16.7%). The UIP group also contained MAAs (*n* = 4, 50%) and ASyS antibodies (*n* = 3, 37.5%). In the OP group, only DM antibodies and ASA antibodies were detected, but no MAAs were detected.

## 4. Discussion

The aim of this study was to carry out an evaluation of patients with positive myositis antibodies and pulmonary involvement in rheumatology. We highlighted which clinical symptoms, examination results and antibodies in our cohort are associated with an increased probability of the occurrence of lung involvement. We were able to show in which rheumatological diagnosis groups the detection of myositis antibodies has less significance regarding the risk of lung involvement. In the group of patients with another IRD, the risk of developing lung involvement corresponds to that of the main diagnosis, such as systemic sclerosis. The additional detection of myositis antibodies does not appear to have any significant diagnostic significance in the risk of lung involvement in other IRD. In the group of patients without an IRD, antibody detection also does not have any important significance regarding the risk of developing lung involvement at the time of the investigation.

Due to the small sample size and the heterogeneous study group, a statistical analysis with the presentation of significance is not sensible. By describing the data, possibilities for prospective larger studies are raised that should be evaluated. An extension of the analysis from laboratory tests in other areas such as neurology or pulmonology using larger datasets could also improve the analysis and possibly prevent bias from the field of rheumatology.

The results of this study emphasize the importance of risk assessment of ILD based on clinical parameters, lung function parameters and myositis antibodies to make the best decisions in diagnostics, like HRCT and therapy.

There is relatively good evidence in the literature regarding the occurrence of lung involvement in connection with anti-Jo-1 antibodies. Therefore, we were limited in analysing patient data for rare ASyS antibodies. We were able to confirm the increased evidence of ILD in the context of PL7 and PL12 antibodies [2]. To ensure data quality for rarer antibodies, the analysis of cases with Jo-1 antibodies was not included. This represents a limitation of this study, because comparison with the Jo-1 group is not possible. However, expansion to include additional centres to enable analysis of anti-HMGCR antibodies would be desirable. For patients who are positive for EJ and OJ antibodies, a valid assessment of the probability of lung involvement in our study is not possible because of the small number of patients included in these antibody groups. With respect to DM-associated antibodies, we were able to confirm the existing evidence regarding MDA5. This approximately corresponds to the other European data. In Asia, this proportion is relatively higher [4]. The described effect of the rather rare occurrence of ILD in connection with Mi-2, NXP-2, SAE and TIF1γ AK could be confirmed in our cohort. The proportion of patients with ILD in the IMNM group is described in the literature as very low, which was also confirmed in our cohort [14]. Although the pathophysiology of the different groups of IMM is not yet fully understood, the existing knowledge regarding the different affected tissues in different IMMs can also be applied to our study results. Pathophysiological studies show that in IMNM, the SRP proteins are located on the muscle cell surface, to which anti-SRP antibodies bind, thus leading to specific histological changes, which do not affect lung tissue. DM and ASyS are more accurately described as multiorgan diseases. The type 1 interferon-inducible genes in DM can be detected in both muscle and lung tissue, which may also explain the high efficacy of JAK inhibitors. The immune response (CD4+ T cells with reactivity against histidyl-tRNA synthetase) in ASyS is also directed against endothelial cells, muscle tissue and lung tissue [2].

Additionally, an increased occurrence of cardiac involvement in IMNM has been described in the literature [2]. In our cohort, we confirmed the cases in the literature with SRP-positive IMNM and PAH [21], although there is not yet much evidence in the literature on this issue [22]. With respect to MAAs, an increased occurrence of ILD has also been described in other studies [7]. However, in our cohort, the proportion of patients with MAAs and evidence of ILD was significantly lower than that in the ASyS antibody group. In the Ku-ab group, a particularly high proportion of PAP values were elevated. This finding is consistent with the existing evidence of the increased occurrence of a Ku-positive overlap syndrome with systemic sclerosis in the literature [18,19,23].

The most frequently described CT pattern in the context of IIM-ILDs is NSIP, which was confirmed in our cohort, in which, in contrast to the literature, the UIP pattern was second, followed by the OP pattern [1,4]. The abovementioned HRCT patterns can also merge into one another over the course of the disease’s history [24]. This represents a limitation of this study, which is due to its retrospective design, as our patients may be in different phases of disease activity. In the literature, OP is more often described in the context of dermatomyositis, which fits with our findings.

In our cohort, the described connection between extrapulmonary symptom complexes such as IIMs related to skin changes, arthritis and Raynaud’s syndrome and the occurrence of ILD was confirmed [4]. Importantly, in our cohort, arthralgias, myalgias or muscle weakness were less strongly associated with ILD. This once again underlines the importance of thorough clinical examination to assess the risk of positive MSAs and MAAs in our patients. Medical history information, such as shortness of breath at rest or during exercise, was particularly strongly associated with pleurisy and PAH. In addition to shortness of breath, symptoms of skin changes and Raynaud’s syndrome were particularly common in the PAH group, which is consistent with findings from the literature [22]. In line with other recent studies, our study revealed that a relevant proportion of patients with ILD evidence on HRCT did not report symptoms of dyspnoea, which highlights the importance of assessing a possible HRCT indication after risk stratification before evidence of dyspnoea.

The major limitations of this study are the retrospective study design and the possible selection bias, which is caused by the monocentric data collection. The lack of longitudinal data also limits its significance. Especially in the group of patients without an existing diagnosis of IRD or myositis antibody detection, long-term observation regarding the possible development of IMM or lung involvement would be important. A more specific statement, which this study does not provide, would be made possible by including histological findings from a muscle biopsy. Regarding the statistical analysis, it can be stated that due to the heterogeneous study group and the rare antibodies, the representation of the investigated variables in percentages is only of limited value given the small number of cases in some subgroups studied.

In summary, the results of this study show for our clinical practice that differentiated assessment of MSAs and MAAs in patients diagnosed with IMM is highly important for the risk stratification of lung involvement. Regarding antisynthetase antibodies, in addition to the Jo-1 antibody, the PL12 antibody is also frequently associated with lung involvement. The MDA5 antibody is the most important myositis antibody with regard to dermatomyositis-associated lung involvement. Clinical parameters such as IIM-specific skin changes, Raynaud’s syndrome or arthritis may be further indications of an increased incidence of lung involvement. In patients treated for another IRD, an additional risk of lung involvement is not to be expected if myositis antibodies are also present. Without clinical or other diagnostic parameters that indicate IMM or another IRD, the determination of myositis antibodies is not useful in determining the risk of lung changes, as longitudinal data are lacking.

Long-term observational studies on the course of possible lung involvement from diagnosis of IIM onwards would be desirable. After a uniform diagnosis, the respective course and occurrence of lung involvement in different IMMs during the course of the disease could be represented depending on the antibody. In addition, we need further randomized controlled trials (RCTs) of therapy for IIM in relation to the different subgroups. Furthermore, using a multicentre registry documenting patients without IRD and with evidence of MSAs or MAAs would be an innovative way to provide new answers. In rheumatology, the approach for patients with ILD-associated myositis antibody detection without IMM is unclear. Long-term observation from the initial antibody detection or reporting of new symptoms could provide new recommendations for possible follow-up in selected constellations.

## Figures and Tables

**Table 1:** Number of patients in the diagnostic groups and the proportion of patients in the diagnostic groups with ILD detected via high-resolution computed tomography (HRCT).

	Number of Patients in the Diagnostic Groups, *n* (%)	Proportion of Patients in the Diagnostic Groups with Radiologic ILD Pattern, *n* (%)
IIM ^1^	37 (17.7)	13 (35.1)
Other IRD ^2^	90 (43.1)	6 (6.7)
No IRD ^3^	82 (39.2)	3 (3.7)

^1^ IIM: idiopathic inflammatory myopathy; ^2^ other IRD: inflammatory rheumatic disease other than IIM; ^3^ no IRD: no inflammatory rheumatic disease; ILD: interstitial lung.

## Data Availability

All the primary data and evaluations on which this study is based are accessible to the investigators at any time in our centre and have been archived for at least 10 years. The datasets presented in this article are not readily available because the data are part of an ongoing study. Requests to access the datasets should be directed to the corresponding author.

## Supplementary Materials

The following supporting information can be downloaded at: https://www.mdpi.com/article/10.3390/jcm14155443/s1, Figure S1. Abnormal examinations and manifestations related to lung involvement in the diagnostic subgroups. The x-axis shows the various tests documented for each patient. The y-axis shows the respective diagnosis subgroup. The numbers of patients within each subgroup are indicated on the right of the diagnosis. The proportion of patients in the various diagnosis subgroups who showed abnormal findings in the respective tests was visualized using a heat map. The numbers in boxes indicate the percentage of patients positive for a particular diagnostic test or manifestation in a diagnostic subgroup. The proportions are color-coded from green (0%) to red (60%). (no IRD: no inflammatory rheumatic disease; ASS: anti-synthetase syndrome; DM: dermatomyositis; PM: polymyositis; OM: overlap-myositis; SSc: systemic sclerosis; SLE: systemic lupus erythematosus; UCTD: undifferentiated connective tissue disease; RA: rheumatoid arthritis; SPA: spondyloarthritis; Other: giant cell arteritis, cryoglobulinemic vasculitis; Behçet’s disease, microscopic polyangiitis, primary Sjogren syndrome, mixed connective tissue disease; ILD: interstitial lung disease; CT: computed tomography; TLC: total lung capacity; FVC: forced vital capacity; DLCO: diffusing capacity of the lungs for carbon monoxide; PAH: pulmonary arterial hypertension).

## Abbreviations

The following abbreviations are used in this manuscript:

ASyS	Antisynthetase syndrome
CAM	Cancer-associated myositis
CTD	Connective tissue disease
DLCO	Diffusion capacity of carbon monoxide
DM	Dermatomyositis
FVC	Forced expiratory vital capacity
HRCT	High-resolution computed tomography
IBM	Inclusion body myositis
IIMs	Idiopathic inflammatory myopathies
ILD	Interstitial lung disease
IMNM	Immune-mediated necrotizing myopathy
IRDs	Inflammatory rheumatic diseases
MAAs	Myositis-associated antibodies
MSAs	Myositis-specific antibodies
NSIP	Nonspecific interstitial pneumonia
OM	Overlap myositis
OP	Organizing pneumonia
PAH	Pulmonary arterial hypertension
PFT	Pulmonary function testing
PM	Polymyositis
RA	Rheumatoid arthritis
SD	Standard deviation
SLE	Systemic lupus erythematosus
SPA	Spondyloarthritis
SSc	Systemic sclerosis
TLC	Total lung capacity
TTE	Transthoracic echocardiography
UCTD	Undifferentiated connective tissue disease
UIP	Usual interstitial pneumonia
