# Supplementary material for: Pulmonary Involvement in Patients with Positive Myositis Antibodies in Rheumatology: A Retrospective Monocentric Analysis
Source: J Clin Med. 2025 Aug 1;14(15):5443. doi: 10.3390/jcm14155443 (PMC12347628; doi:10.3390/jcm14155443)
Supplement: Supplementary file 1 [file jcm-14-05443-s001.zip › jcm-3744238-supplementary.pdf]

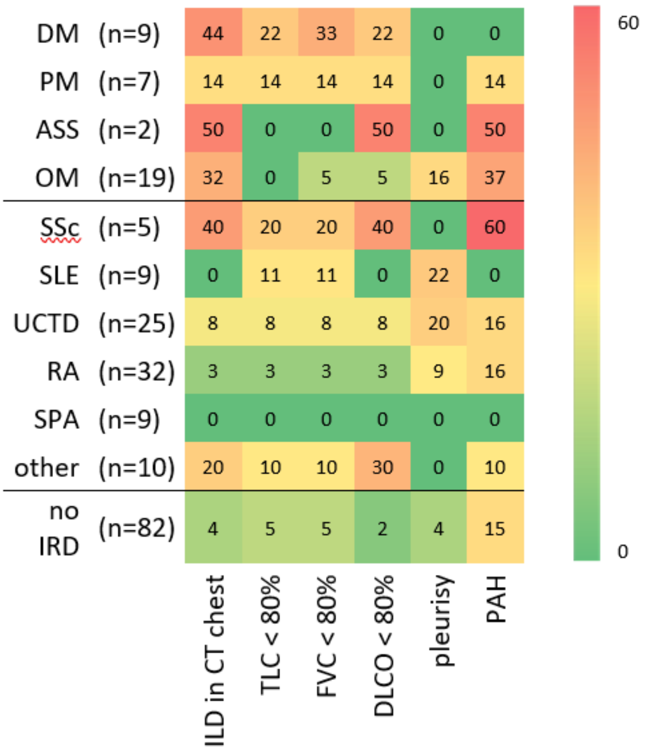

Figure S1. *Abnormal examinations and manifestations related to lung involvement in the diagnostic subgroups. The x-axis shows the various tests documented for each patient. The y-axis shows the respective diagnosis subgroup. The numbers of patients within each subgroup are indicated on the right of the diagnosis. The proportion of patients in the various diagnosis subgroups who showed abnormal findings in the respective tests was visualized using a heat map. The numbers in boxes indicate the percentage of patients positive for a particular diagnostic test or manifestation in a diagnostic subgroup. The proportions are color-coded from green (0%) to red (60%). (no IRD: no inflammatory rheumatic disease; ASS: anti-synthetase syndrome; DM: dermatomyositis; PM: polymyositis; OM: overlap-myositis; SSc: systemic sclerosis; SLE: systemic lupus erythematosus; UCTD: undifferentiated connective tissue disease; RA: rheumatoid arthritis; SPA: spondyloarthritis; Other: giant cell arteritis, cryoglobulinemic vasculitis; Behçet's disease, microscopic polyangiitis, primary Sjogren syndrome, mixed connective tissue disease; ILD: interstitial lung disease; CT: computed tomography; TLC: total lung capacity; FVC: forced vital capacity; DLCO: diffusing capacity of the lungs for carbon monoxide; PAH: pulmonary arterial hypertension).*
